# Altered CXCR3 isoform expression regulates prostate cancer cell migration and invasion

**DOI:** 10.1186/1476-4598-11-3

**Published:** 2012-01-11

**Authors:** Qian Wu, Rajiv Dhir, Alan Wells

**Affiliations:** 1 Department of Pathology, University of Pittsburgh, Pittsburgh, Pennsylvania, 15261, USA; 2 Pittsburgh VAMC, Pittsburgh, Pennsylvania, 15213, USA

**Keywords:** prostate cancer, CXCR3, cell migration, invasion, calpain

## Abstract

**Background:**

Carcinoma cells must circumvent the normally suppressive signals to disseminate. While often considered 'stop' signals for adherent cells, CXCR3-binding chemokines have recently been correlated positively with cancer progression though the molecular basis remains unclear.

**Results:**

Here, we examined the expression and function of two CXCR3 variants in human prostate cancer biopsies and cell lines. Globally, both CXCR3 mRNA and protein were elevated in localized and metastatic human cancer biopsies compared to normal. Additionally, CXCR3A mRNA level was upregulated while CXCR3B mRNA was downregulated in these prostate cancer specimens. In contrast to normal prostate epithelial cells (RWPE-1), CXCR3A was up to half the receptor in the invasive and metastatic DU-145 and PC-3 prostate cancer cells, but not in the localized LNCaP cells. Instead of inhibiting cell migration as in RWPE-1 cells, the CXCR3 ligands CXCL4/PF4 and CXCL10/IP10 promoted cell motility and invasiveness in both DU-145 and PC-3 cells via PLCβ3 and μ-calpain activation. CXCR3-mediated diminution of cell motility in RWPE-1 cells is likely a result of cAMP upregulation and m-calpain inhibition via CXCR3B signal transduction. Interestingly, overexpression of CXCR3B in DU-145 cells decreased cell movement and invasion.

**Conclusion:**

These data suggest that the aberrant expression of CXCR3A and down-regulation of CXCR3B may switch a progression "stop" to a "go" signal to promote prostate tumor metastasis via stimulating cell migration and invasion.

## Introduction

Prostate cancer is the most frequently diagnosed cancer and a leading cause of cancer death in men, with the mortality and morbidity being mainly due to tumor invasion and metastasis [1]. Current therapies are only effective against localized prostate cancer; once the tumor invades and disseminates to surrounding tissues or metastasizes to distance sites, current treatments only slightly prolong patient survival [1-4]. Thus, patient benefit awaits rational approaches targeting the molecular underpinnings of this transition to tumor dissemination.

Tumor invasion and metastasis requires, among other cell behaviors, enhanced cancer cell motility [5-10]. Many studies have found that invasive prostate cancer cells have enhanced motility in response to paracrine, autocrine and matrix-derived pro-migratory signals [10-14]. Thus, these signals and the receptors and intracellular signaling pathways through which they actuate motility represent potential targets. However, the myriad such factors and numerous pathways make this type of 'attenuative' approach difficult and/or short-lived.

A novel potential approach to limit tumor dissemination would be to re-instate the physiological 'stop' signals that keep normal and dysplastic epithelial cells localized. Work in this area has mainly focused on downregulation of cell-cell adhesion molecules such as E-cadherin during the acquisition of EMT or upregulation of matrix metalloproteinases [10,12,15,16]. More recently, paracrine signals have been recognized as providing additional inhibition to migration. The family of chemokines that bind to the CXCR3 receptor has been shown to inhibit the motility of adherent cells such as fibroblasts and endothelial cells, even while being chemotactic for leukocytes [17-19].

CXCR3, a receptor for ELR-negative CXC chemokines, is activated by specific binding of the ligands, CXCL4/PF4, CXCL9/MIG, CXCL10/IP10, CXCL11/IP9/I-TAC, resulting in diverse cellular responses, including chemotactic migration and cell proliferation, or inhibition of migration and even endothelial death depending on the cell type [20]. This diversity of cell behaviors is explained, in part, by the presence of two splice variants of CXCR3, CXCR3A and CXCR3B; CXCR3B contains a longer extracellular domain at the N-terminus [19]. CXCR3A mainly functions in the chemotactic activity on activated T lymphocytes and Natural Killer (NK) cells [21,22]. Additionally, CXCR3A has also been shown to promote cell proliferation [19]. However, CXCR3B, primarily found expressed on fibroblasts, endothelial and epithelial cells, inhibits cell migration and endothelial apoptosis [18,19]. Some studies have suggested that CXCR3A and CXCR3B play reciprocal roles through different G-protein coupling and trigger distinct signaling transduction pathways [19,23,24], though there is some evidence for overlap in signaling cascades with differential cellular outcomes being the integration of signaling and the cell milieu [25,26]. Thus, differential responsiveness of carcinoma cells may be due to either the cellular milieu or the CXCR3 isoform presentation.

CXCR3 expression is ubiquitous, though regulated in some cell types. Interestingly, increased express has been shown to positively correlate with human breast, colon, renal, and prostate cancer [27-35]. Several groups have reported that CXCR3 expression is linked to breast, colon, osteosarcoma and melanoma cell metastasis by regulating cell proliferation and/or cell migration in murine models [31,34-38]. However, these studies did not account for isoform usage since the CXCR3B isoform was identified only recently, and isolated detection of CXCR3A is difficult due to almost complete overlap with CXCR3B. A hint that the isoform distribution may be important in tumor progression was provided by a study in renal carcinomas in which treatment with calcineurin inhibitors results in bigger tumors in nude mice secondary to downregulation of CXCR3B; in actuality increased CXCR3B expression correlates with tumor necrosis in renal cell carcinoma [30,32]. This may indicate that the isoform normally expressed on epithelial cells, CXCR3B, can be a tumor suppressive signal. However, these data also call for a more nuanced understanding of CXCR3 signaling in carcinoma progression, to clarify the seemingly contradictory findings.

Herein, we dissect CXCR3 functioning in prostate carcinomas and derived cell lines. Our studies for the first time demonstrated that both CXCR3 mRNA and protein expression was upregulated in human localized prostate cancer and metastatic prostate cancer. More importantly, CXCR3 splice variants exhibited different mRNA expression profile-CXCR3A mRNA level was high and CXCR3B mRNA was low in prostate cancer compared to normal prostate. In addition, CXCR3B, the dominant CXCR3 splice variant in normal prostate epithelial cells (RWPE-1), was replaced in part by CXCR3A in invasive and metastatic prostate cancer cell lines (DU-145 and PC-3) and promoted cell motility and invasiveness *in vitro*. This increase of prostate cancer cell migration and invasion was not only a result of PLCβ3 activation by CXCR3A, but also required downregulation of the strength of inhibitory signal via CXCR3B. Restoring higher CXCR3B expression in DU-145 cells significantly blocked CXCR3-chemokine-induced cell movement and invasion. These in vitro findings suggest that the aberrant expression of CXCR3A and down-regulation of CXCR3B play an important role in promoting prostate tumor invasion and metastasis via subverting an anti-migratory to a pro-migratory signal.

## Results

### CXCR3 and its splice variant expression in human prostate carcinoma tissues

**Figure 1:**
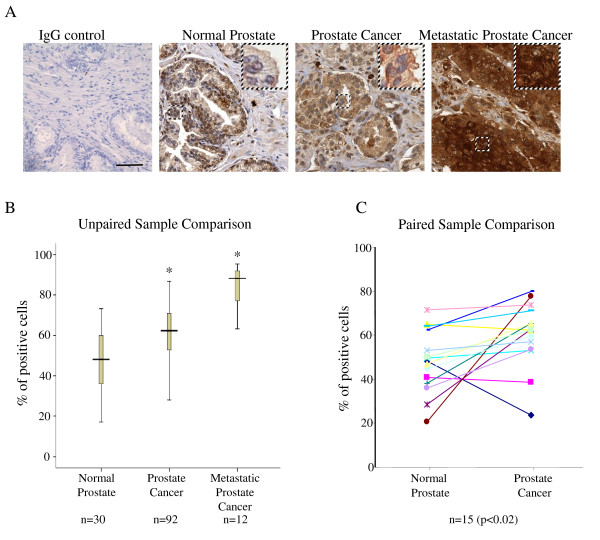
**CXCR3 expression and localization in prostate tumor tissues**. (A) CXCR3 localization in human normal prostate, localized and metastatic prostate cancer. The insets are at higher magnification to show the cellular localization of CXCR3 in the boxed areas. Pictures are representative among the samples. Bar: 100 μm. (B) and (C) CXCR3 was upregulated in human localized and metastatic prostate cancer tissue. (B) Box plot of CXCR3 expression in human normal prostate, localized and metastatic prostate cancer by percentage of CXCR3-positive cells (*P < 0.01). (C) Quantification of CXCR3 expression in paired normal and cancerous prostate samples (P < 0.02).

**Figure 2:**
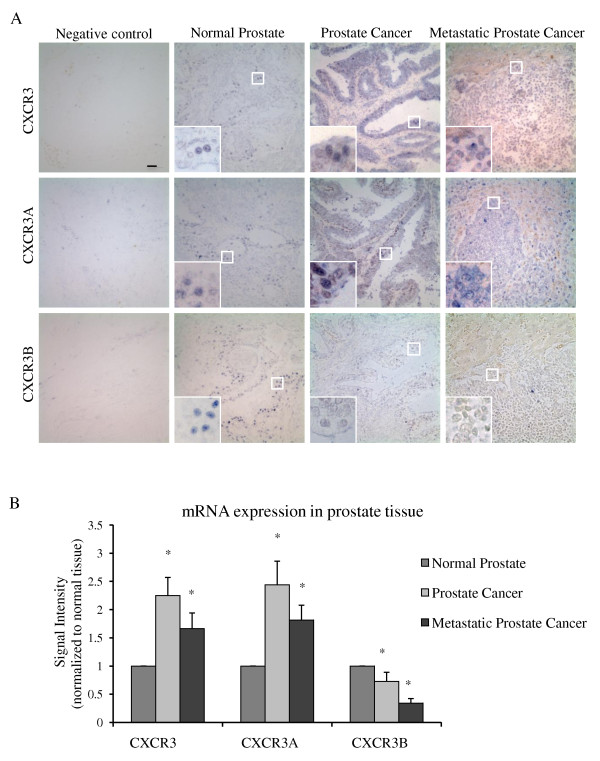
**mRNA expression of CXCR3 isoforms in prostate tumor tissues**. (A) CXCR3, CXCR3A and CXCR3B mRNA expression in human normal prostate, localized and metastatic prostate cancer. The inserts are the high magnification pictures. Bar: 10 μm. (B) Quantification of CXCR3, CXCR3A andCXCR3B mRNA expression as shown in human normal prostate, localized and metastatic prostate cancer. Signal intensity was measured by imageJ and the signal intensities of normal tissues were set as 1 in each group.

To study CXCR3 expression in human prostate carcinomas, a human tissue microarray was generated with samples from the University of Pittsburgh Tumor Tissue Bank. Thirty (30) normal prostate tissue, 92 prostate cancer tissue and 12 metastatic prostate cancer tissue were analyzed. In normal prostate tissue, CXCR3 was primarily expressed in all gland epithelial cells and in some stromal cells (Figure 1A). In primary prostate cancer samples, somewhat upregulated CXCR3 staining was observed which was quantified by the percentage of positive-stained cells (Figure 1B). This result was further confirmed by paired-sample comparison (Figure 1C). An even higher percentage of positive cells was markedly seen in metastatic prostate cancer tissue (Figure 1B). However, in a survey across an admittedly limited number of specimens, the increases in CXCR3 expression appeared to be independent of the target organ of the metastases (Additional file 1). Examining single cells, CXCR3 was predominantly on the cell membrane in normal prostate tissue and primary carcinomas but this localization was replaced with a whole cell stain in metastatic prostate cancer tissue (Figure 1A, enlarged boxes). Additionally, the result from in situ hybridization targeting CXCR3 in 5 normal prostate, 6 localized prostate cancer and 6 metastatic prostate cancer samples showed that CXCR3 mRNA expression significantly upregulated in localized and metastatic prostate cancer patients (Figure 2), which was consistent with CXCR3 protein expression profile in prostate cancer.

The two splice isoforms of CXCR3 have been reported to play different roles in cellular function regulation; therefore, CXCR3A and CXCR3B expression patterns were examined in human prostate by in situ hybridization (Figure 2A). Interestingly, CXCR3A mRNA was increased while CXCR3B mRNA was decreased in the prostate cancer samples compared to normal prostate controls (Figure 2B), suggesting that the switch of CXCR3 isoform expression may play an important role in prostate cancer dissemination, invasion and metastasis.

### Prostate carcinoma cell lines express CXCR3A in contrast to normal prostate epithelial cells

**Figure 3:**
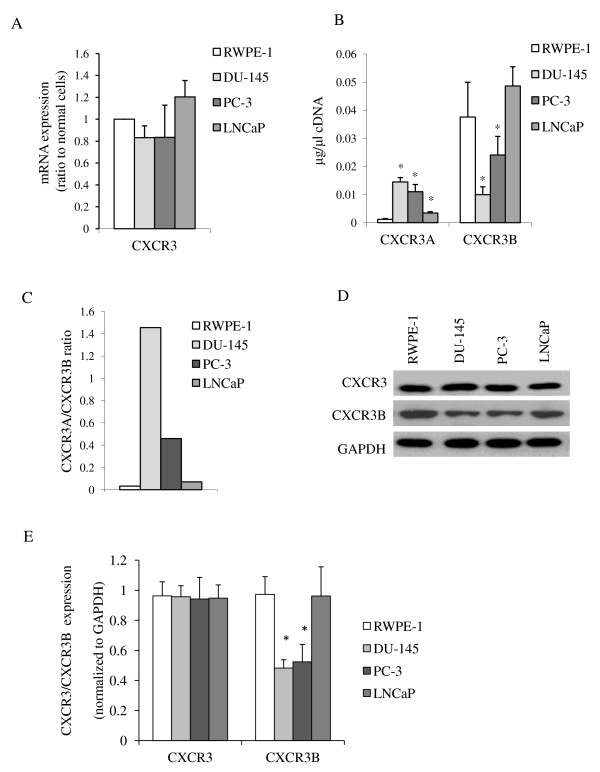
**CXCR3 expression in human normal and prostate cancer cell lines**. (A) mRNA expression of CXCR3 in RWPE-1, DU-145, PC-3 and LNCaP cells. CXCR3 RNA was normalized to GAPDH RNA levels for that cell line, prior to normalizing to RWPE-1 cells. Histograms represent mean values (+/-s.d.) of more than three separate experiments. (B) mRNA expression of CXCR3A and CXCR3B in RWPE-1, DU-145, PC-3 and LNCaP cells. The concentration was calculated by a standard curve using known amounts of CXCR3A or CXCR3B plasmids. Histogram represent mean values (+/-s.d.) of more than three separate experiments (*P < 0.05 compared to RWPE-1). (C) Ratio of CXCR3A vs. CXCR3B mRNA expression. The calculation was based on the data in (B). DU-145 and PC-3 cells showed higher ratio than RWPE-1 cells. (D) Protein expression of total CXCR3 and CXCR3B in RWPE-1, DU-145, PC-3 and LNCaP cells. (E) Quantification of protein expression in prostate cancer cells. Histogram represent mean values (+/-s.d.) of three separate experiments (*P < 0.05 compared to RWPE-1).

To study CXCR3 and its splice variant function in prostate cancer, CXCR3 expression was first examined in three commonly studied prostate cancer cell lines, DU-145, PC-3 and LNCaP. DU-145 and PC-3 cell lines are both androgen-insensitive invasive and metastatic in murine xenograft models while LNCaP is androgen-sensitive and remains localized upon orthotopic inoculation, even though all were derived from prostate cancer metastases. Compared to normal prostate epithelial cells (RWPE-1), all tested prostate cells expressed similar level of total CXCR3 at both mRNA and protein levels (Figure 3A, D and 3E). Looking at the CXCR3 splicing isoform expression, in contrast to RWPE-1 cells, in which CXCR3B was basically the only splice variant, both CXCR3A and CXCR3B were expressed at near equivalent levels in the two invasive and metastatic prostate cancer cell lines, DU-145 and PC-3, but not in the LNCaP cells (Figure 3B and 3C). As a result, CXCR3B protein expression reduced to approximately 50% in DU-145 and PC-3 cells compared to RWPE-1 cells (Figure 3D and 3E).

As epithelial cells can express the CXCR3-binding chemokines, we queried for potential autocrine stimulatory loops. RNA and protein levels of two known ligands of CXCR3, CXCL10/IP10 and CXCL11/IP9 were down-regulated in the tumor lines. CXCL4/PF4 was up-regulated in DU-145 and PC-3 cells but not in LNCaP cells (Additional file 2). Another ligand CXCL9/MIG showed overall negligible levels of mRNA expression.

CXCR3 is a seven-transmembrane receptor, whose localization plays a key role in its activity. The cellular localizations of CXCR3 and CXCR3B were examined in RWPE-1, DU-145, PC-3 and LNCaP cells by flow cytometry, in which CXCR3 or CXCR3B proteins were labeled by specific antibodies with or without prior cell permeabilization; these detections represent total protein and membranous protein, respectively (Additional file 3) (examination of the CXCR3A isoform is not currently possible as there are no available antibodies that are sufficiently specific for CXCR3A due to near complete overlap in sequence). The fluorescence-positive cells revealed both CXCR3 or CXCR3B were more abundant in the cytosolic location in DU-145 and PC-3 as opposed to surface locale in RWPE-1 and LNCaP cells, which is similar to the CXCR3 localization in human metastatic prostate carcinoma tissues (Figure 1A enlarged boxes). This suggests that CXCR3/CXCR3B internalization and turnover might be occurring in advanced prostate carcinoma cells, indicative of auto- and para-crine stimulation.

### CXCR3-chemokine-induced cell motility and invasion is elevated in prostate cancer cells via PLCβ3 signaling pathway

**Figure 4:**
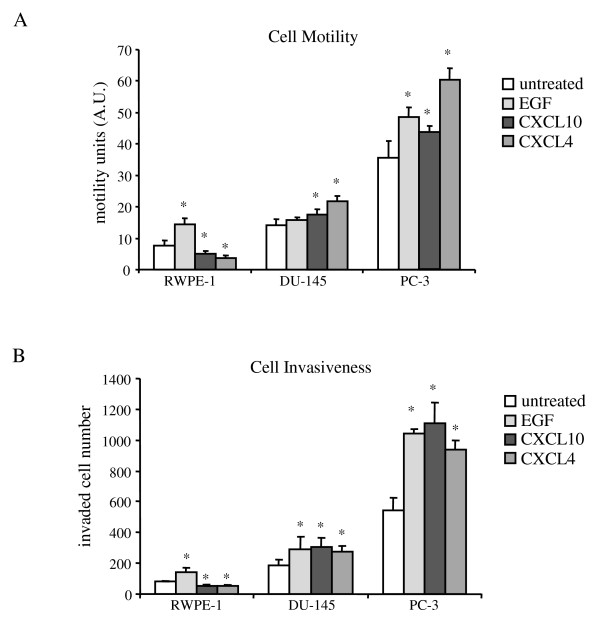
**CXCR3 chemokine promoted prostate cancer cell motility and cell invasion**. (A) cell motility and (B) cell invasiveness in RWPE-1, DU-145 and PC-3 cells after 5 nM EGF or 1 μg/ml chemokine treatment for 16 hrs. The moving distance (arbitrary units, A.U.) in cell motility assay was measured by the pixel changes in ImageJ after 16 hrs. Cell invasiveness was evaluated by the number of invaded cells after 16 hr treatment in Matrigel invasion chamber system. Histogram represent mean values (+/-s.d.) of more than three separate experiments each in triplicate (*P < 0.05).

With the above data linking CXCR3 upregulation to prostate cancer progression and the switch to expressing both isoforms, we queried how this affects cell behaviors [18,25,39]. Even though CXCR3 has been reported as a cell growth regulator in select cancers [37,38,40], CXCR3-chemokines did not alter the cell proliferation in the prostate cancer lines tested (data not shown). Therefore, we looked at cell motility induced by CXCR3 signal transduction. Since CXCL4/PF4 and CXCL10/IP10 represent the main CXCR3 ligands found during platelet degranulation and thus any hemorrhage and deep in reactive/wounded stromal compartment respectively, we examined functions of these two CXCR3 chemokines on prostate carcinoma cell functioning. Due to low basal and growth factor stimulated cell motility and invasiveness (data not shown), LNCaP cells were not used for chemokine-induced cell motility and invasion examination in the following studies. As expected, CXCR3 ligands inhibited cell motility in RWPE-1 cells. Interestingly, CXCL4/PF4 and CXCL10/IP10 promoted cell motility in both DU-145 and PC-3 cells *in vitro *(Figure 4A). CXCR3 blocking antibodies prevented chemokines-induced cell motility significantly in DU-145 cells suggesting that cell motility was induced specifically through CXCR3 (Additional file 4). Since cancer cell motility is tightly related to cancer invasion, we next examined DU-145 and PC-3 invasiveness in a CXCR3-chemokine environment. Unsurprisingly, the CXCR3 chemokines blocked RWPE-1 cell invasion through a Matrigel matrix barrier, but increased the invasiveness of both prostate cancer lines (Figure 4B). These data suggest that activated CXCR3 signaling may drive prostate cancer cells invasion and metastasis.

**Figure 5:**
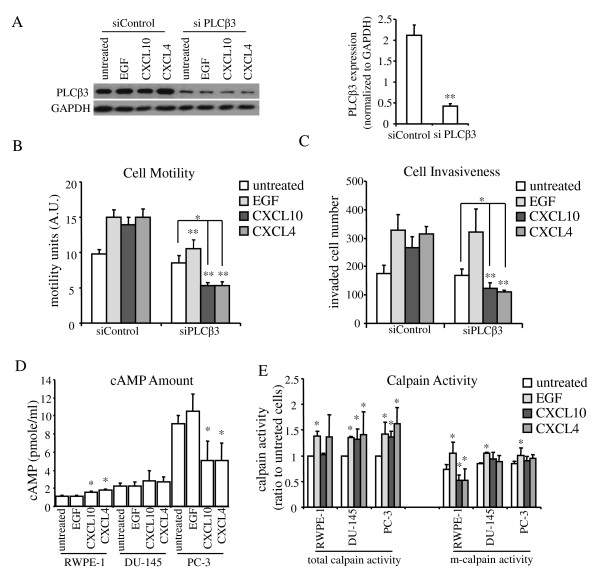
**CXCR3 chemokine induced cell motility and invasion via PLCβ3 signaling pathway in prostate cancer cells and blocked cell motility by m-calpain activity inhibition in normal cells**. (A) PLCβ3 was significantly knocked down in DU-145 cells by siRNA. Protein expression was quantified by ImageJ and normalized to GAPDH. Histogram represent mean values (+/-s.d.) of three separate experiments (**P < 0.05). CXCR3-chemokine-induced (B) cell motility and (C) cell invasiveness in DU-145 cells reduced after PLCβ3 downregulation. Histogram represent mean values (+/-s.d.) of three separate experiments (*P < 0.05, compared to untreated controls; **P < 0.05, compared to siControl group). (D) cAMP amount in RWPE-1, DU-145 and PC-3 cells after chemokine induction. Histogram represent mean values (+/-s.d.) of three separate experiments (*P < 0.05 compared to untreated controls). (E) Calpain activity in RWPE-1, DU-145 and PC-3 cells after chemokine treatment. Calpain activity was quantified by fluorescent intensity of calpain substrate. The signal intensities of untreated samples were set as 1 in each group. Histogram represents mean values (+/-s.d.) of three separate experiments (*P < 0.05 compared to untreated controls). Total calpain activity included both μ-calpain and m-calpain.

CXCR3 is a G-protein-coupled receptor and the two different isoforms appear to activate different downstream signaling pathways. CXCR3A and CXCR3B both activate PLCβ and induce downstream intracellular Ca^++ ^flux, which activates μ-calpain to loosen cell-substratum adhesion and promote cell motility. CXCR3B signaling also triggers PKA, known as cAMP-dependent protein kinase, which in turn inhibits m-calpain activation, preventing tail release and blocking cell migration [18,19,24,25,41-43]. We had previously shown that inhibiting m-calpain limits prostate cancer cell invasion and metastasis in xenograft models as well as in vitro [44,45]. To dissect which signaling pathway was dominant in prostate cancer cells leading to cell migration, we queried these intermediaries. Firstly, as there are many isoforms of PLCβ, PLCβ3 was chosen due to its predominant expression in the prostate cell lines (data not shown). PLCβ3 protein expression was reduced to a quarter of its level by siRNA in DU-145 cells as the test line (Figure 5A). With markedly reduced PLCβ3 expression, CXCR3-mediated cell motility and invasiveness both decreased dramatically in DU-145 cells, suggesting that CXCR3 promoted cell migration and invasion through PLCβ3 pathway (Figure 5B and 5C). Furthermore, when CXCR3B was downregulated by siRNA transfection in DU-145 cells without affecting CXCR3A expression, no changes of cell motility were observed (Additional file 5), indicating that the activation of cell migration was mainly a result of PLCβ3 activity through CXCR3A signaling pathway in DU-145 cells.

### Inhibition of cell motility and invasion in normal prostate cells correlated with m-calpain activity blockage

To examine whether the cell motility inhibitory signal pathway(s) via CXCR3B is active or not in normal and cancerous prostate cells, cAMP was analyzed after ligand exposure. Prostate cancer cells showed higher cAMP at an overall level than normal cells. In RWPE-1 cells, CXCL4/PF4 and CXCL10/IP10 markedly elevated cAMP amount. In contrast, neither of these two CXCR3 chemokines changed the elevated cAMP abundance in DU-145 cells but reduced to some extent the very elevated levels in PC-3 cells (Figure 5D); however, this was on the background of greatly elevated basal cAMP making changes in levels less relevant than absolute levels which were higher than even stimulated levels in RWPE-1 cells. Since μ-calpain and m-calpain are regulated by PLCβ/Ca^++ ^and cAMP/PKA pathways respectively, which play direct and essential roles in cell migration regulation, we next examined calpain activities in these cells. Total calpain activity (from both μ-calpain and m-calpain) did not change much in RWPE-1 cells after CXCR3 chemokine treatment. Interestingly, m-calpain activity significantly decreased with CXCL4/PF4 and CXCL10/IP10 in these normal prostate cells (Figure 5E), suggesting that inhibition of cell motility and invasiveness from CXCR3 chemokines is a result of m-calpain activity reduction. More importantly, this activity decrease was not a result of m-calpain protein expression level change (Additional file 6). Since there is no increase of cAMP amount after CXCL4/PF4 or CXCL10/IP10 treatment in the prostate cancer cell lines, m-calpain activities remained at same levels compared to the untreated cells (Figure 5E), suggesting that inhibition of cell migration via the CXCR3B pathway was not active in prostate cancer cells.

### CXCR3B overexpression in DU-145 cells blocked chemokine-induced cell motility and invasion via m-calpain activation inhibition

**Figure 6:**
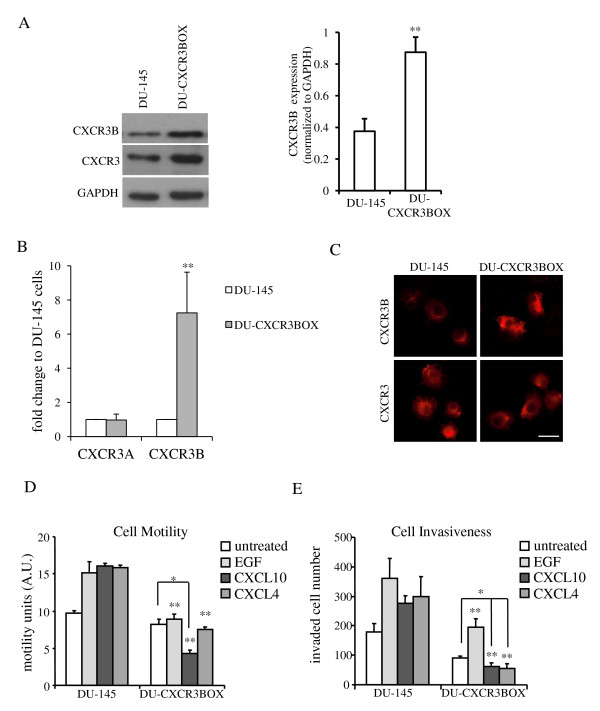
**CXCR3B overexpression in DU-145 cells blocked chemokine-induced cell motility and invasion**. (A) CXCR3 and CXCR3B protein expression in DU-145 and CXCR3B overexpression cells (**P < 0.05, compared to DU-145). The expression was quantified by ImageJ and normalized to GAPDH. (B) CXCR3A and CXCR3B mRNA expression in DU-145 and CXCR3B overexpression cells (**P < 0.05, compared to DU-145). This showed no CXCR3A mRNA upregulation in CXCR3B overexpression cells. (C) CXCR3B localization after CXCR3B plasmid expression in DU-145 cells. Bar: 20 μm. CXCR3-chemokine-induced (D) cell motility and (E) cell invasiveness in DU-145 cells decreased after CXCR3B expression. Same methods and analyses were used as in Figure 3. Histogram represent mean values (+/-s.d.) of three separate experiments (*P < 0.05, compared to untreated controls; **P < 0.05, compared to DU-145 group).

CXCR3B was found to be the primary CXCR3 isoform in prostate normal tissue and prostate epithelial RWPE-1 cells. However, in prostate carcinoma tissues and cell lines, not only was CXCR3A highly expressed but the level of CXCR3B was reduced. Thus, a question remains as to whether the reduced expression of CXCR3B was operative rather than the novel expression of CXCR3A. To understand better about CXCR3B signaling in prostate cancer cells, the CXCR3B splice variant was overexpressed in DU-145 cells up to 2 fold at the protein expression level (Figure 6A). Overexpression of CXCR3B in DU-145 cells did not change CXCR3A or CXCR3-ligands expression levels at a mRNA level or cellular localization of CXCR3 (Figure 6B, C and data not shown). No proliferation rate alteration was observed in these cells either (data not shown). However, in these DU-145 cells with CXCR3B overexpression, chemokines inhibited cell motility and invasion (Figure 6D and 6E), suggesting that prostate cancer cell motility and invasiveness elevation was due to a lack of CXCR3B signaling at least in part in addition to CXCR3A expression.

**Figure 7:**
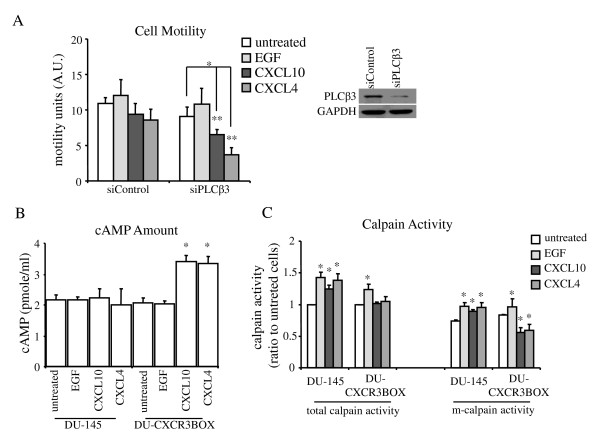
**CXCR3B upregulation in DU-145 cells inhibited m-calpain activity**. (A) Cell motility reduced in DU-145 CXCR3 overexpressing cells after PLCβ3 downregulation. Histogram represent mean values (+/-s.d.) of three separate experiments (*P < 0.05, compared to untreated controls; **P < 0.05, compared to siControl group). Western blot was representative for three separate experiments. (B) cAMP amount increased after chemokine treatment in DU-CXCR3BOX cells but not DU-145 cells. Histogram represent mean values (+/-s.d.) of three separate experiments (*P < 0.05 compared to untreated controls). (C) Calpain activity in DU-145 and DU-CXCR3BOX cells after chemokine treatment. Same methods and analyses were used as in Figure 4E. Histogram represent mean values (+/-s.d.) of three separate experiments (*P < 0.05 compared to untreated controls). Total calpain activity included both μ-calpain and m-calpain.

However, to examine whether CXCR3 expression still contributes to motility, PLCβ3 was down-regulated by siRNA and cell motility was measured. Interestingly, DU-145 cells with CXCR3B overexpression and PLCβ3 knockdown showed a further reduction of cell motility compared to cells with CXCR3B overexpression only (Figure 7A), suggesting that PLCβ3 was still active in DU-145-CXCR3BOX cells, but that CXCR3 signaling through PLCβ3 was contributing positively to migration; this might be occurring through an endogenous CXCR3A signal. We observed that cell motility and invasion was inhibited in both RWPE-1 and DU-CXCR3BOX prostate cancer cells, and this inhibition is due to upregulation of cAMP level and m-calpain activity reduction in RWPE-1 cells (Figure 4 and 6). Therefore, we asked the question that whether DU-CXCR3BOX cells activated same signaling pathway mainly through CXCR3B to block cell motility and invasiveness. Unsurprisingly, CXCL4/PF4 and CXCL10/IP10 treatment increased cAMP levels in DU-CXCR3BOX cells (Figure 7B), which blocked m-calpain activity (Figure 7C). These data indicate that even in the face of promigratory signaling from CXCR3A, high levels of CXCR3B signaling can override this to inhibit cell movement.

## Discussion and conclusion

Our findings, for the first time, illustrate that CXCR3 isoform switching may impact tumor dissemination, shifting a usually anti-migratory 'stop' signal into a pro-migratory and invasive "go" signal. Previous studies had identified distinct signaling pathways by which this receptor could actuate diametrically opposite motility behaviors. Both CXCR3A and CXCR3B activate PLCβ downstream of heterotrimeric G proteins. PLCβ hydrolyzes phosphatidylinositol 4,5-bisphosphate (PIP2), generating two products: inositol 1,4,5-trisphosphate (IP3), a universal calcium mobilizing second messenger; and diacylglycerol (DAG), an activator of protein kinase C (PKC). IP3 induces intracellular Ca^++ ^flux, which activates μ-calpain and results in cell motility induction by lessening adhesion [25]. PKC plays a positive role in ERK activation and followed by m-calpain phosphorylation and activation. In a word, CXCR3 signals from PLCβ activity promotes cell migration unless the cell detaches due to the cleavage of a predominant β3 integrin as in endothelial cells [46]. A unique signal transduction path via CXCR3B leads to an accumulation of cAMP. With CXCR3B signals, PKA, known as cAMP-dependent protein kinase, is activated which inhibits m-calpain activation and blocks cell migration [18,19,24,25,41-43]. Thus, the cell outcome is determined by the balance between these two pathways and the cell's overall adhesiveness and complement of integrins.

The findings in tissue and epithelial cells (RWPE-1) suggest that CXCR3B is the dominant splice variant in normal human prostate tissues and these cells. CXCL4/PF4 and CXCL10/IP10 inhibited RWPE-1 cell motility and invasiveness mainly via cAMP upregulation and m-calpain activity reduction through CXCR3B. In these normal cells, PLCβ3 was still active because intracellular Ca^++ ^flux could be induced and total calpain activity increased, suggesting that CXCL10/CXCL4-CXCR3B axis also turned on pro-migratory signals. However, μ-calpain and m-calpain activity are both required for cell motility as they act at distinct site in the cell [47]; hence, inhibiting m-calpain to prevent rear de-adhesion blocked RWPE-1 migration and invasion and was dominant over the de-adhesion-mediated motility.

In invasive and metastatic prostate cancer cells (DU-145 and PC-3), CXCR3A and CXCR3B are both expressed with CXCR3B being reduced in level compared to the normal prostate cell line. CXCR3 ligands, CXCL10/IP10 and CXCL11/IP9 were downregulated in all tested prostate cancer cells and CXCL4/PF4 were elevated in DU-145 and PC-3 cells (Additional file 2). These ligand expression data suggest that CXCL10/IP10 and CXCL11/IP9 might be an operative ligand in normal prostate cells, while CXCL4/PF4 may play a role in the invasive and metastatic cells, though definitive testing of such awaits further testing. Our data revealed that CXCL4/PF4 and CXCL10/IP10 both promoted migration and invasiveness *in vitro *in prostate cancer cells. This motility was blocked by CXCR3 antibody significantly and CXCR3B antibody mildly in DU-145 cells (Additional file 4 and data not shown), indicating that cell motility activation in prostate cancer cells was due mainly to CXCR3A but that CXCR3B may also contribute. We must note that Lasagni et al. reported CXCR3B isoform in microvascular endothelial cells and suggested CXCL4/PF4 is a CXCR3B-specific ligand [19]. However, other later work suggests CXCL4/PF4 induces activated T lymphocytes migration through CXCR3A signaling [26]. In any case at the higher levels of ligand, CXCL4/PF4 appears to activate both isoforms. In DU-145 and PC-3 cells, cAMP activity was sustained at a high level and no further upregulation of cAMP was able to be detected by any CXCR3 chemokine treatment, resulting in no inhibition of m-calpain via CXCR3B pathway. This high level of cAMP is correlated with upregulated PKA activity in DU-145 and PC-3 cells compared to RWPE-1 cells (Additional file 7), and thus is likely not further activated by CXCR3B signaling. In summary, in these prostate cancer cells, PLCβ3 plays an essential role on cell migration promotion which may be through μ-calpain activation. However, CXCR3B-induced inhibitory signals were not effective.

We then queried whether the key change was expression of CXCR3A or also a quantitative decrement in CXCR3B. When exogenous CXCR3B was expressed in DU-145 to bring the balance of CXCR3 isoform back, even higher than RWPE-1 cells, cell motility and invasiveness decreased, recapitulating the behavior of RWPE-1 cells (this was confirmed in two distinct individual colons). The inhibition in these DU-145-CXCR3BOX cells is a result of increased cAMP after CXCR3 chemokine induction, following by m-calpain activity inhibition, which is the same pathway that limits dissemination in RWPE-1 cells. The migratory effects of CXCR3 isoform signaling in LNCaP cells would be of interest but as the basal motility levels of these cells is very low, this line of investigation is not productive. Based on the analysis of CXCR3 ligand expression in LNCaP, very low levels of all the ligands suggest the CXCR3 signaling activation may not be an essential role in cell migration regulation in this line. The other aspect, downregulation of CXCR3A to restore a quantitative excess of CXCR3B was not accomplished as the complementary molecules to downregulate this isoform would also recognize the CXCR3B mRNA. Even in the absence of this validation, the regulation of the balance of CXCR3 splicing variants still could be a key factor for prostate cancer to become motile and invasive. The differences of CXCR3 receptor and ligand expression in various prostate cancer cell lines might be a result from metastatic organ specificity [38]; however, immunohistochemistry analysis of a limited set of prostate metastases indicated that CXCR3 expression is not organ selective at least to a significant degree (Additional file 1). The integrity and heterogeneity of CXCR3 expression and regulation in cancer require further investigation.

It remains to be determined whether matrix remodeling, in addition to motility alteration, regulates invasiveness in response to CXCR3 signaling. As an initial examination of matrix alterations, we checked MMP2 and MMP9 expression levels, which have been shown to be regulated by CXCR3 signals [12,48]. Interestingly, RWPE-1 cells exhibited the highest levels of MMPs among the tested cells and both MMP2 and MMP9 RNA levels were almost negligible for the prostate cancer cells (Additional file 8A). With CXCL4 and CXCL10 treatment, MMP2 expressions dramatically increased in RWPE-1 and LNCaP cells; however, even with increase, MMP2 expressions in LNCaP cells were still low. MMP9 was mainly upregulated in PC-3 and LNCaP cells but this increase could be negligible due to a low absolute expression (Additional file 8B). These data suggest CXCR3-induced MMP elevation may not play a critical role in the regulation of prostate cancer cell motility. This is consistent with our earlier findings that while matrix proteases were required for DU-145 invasiveness in vitro and dissemination in vivo [49,50], their regulation was not a major regulator of these properties.

Our results from in vivo studies found that more cells in localized and metastatic prostate tumors expressed CXCR3 compared to normal prostate tissue (Figure 1). Interestingly, this upregulation of CXCR3 was also observed in breast cancer wherein it was correlated to poor patient survival [31], suggesting that CXCR3 could be an important pro-dissemination signal for cancer dissemination, invasion and metastasis. Primary localization of CXCR3 in normal prostate tissues was membranous. In contrast, CXCR3 seems to have relocalized from the cell membrane to the cytosol in prostate tumors, as was also detected in tissue-cultured cell lines (Figure 1 and Additional file 3); this could reflect internalization/downregulation based on autocrine/paracrine signaling or hint at a distinct signaling function from intracellular organelles. Other than prostate epithelial cell expressing CXCR3, some prostate stromal cells as well as endothelial cells also showed CXCR3 expression in prostate cancer tissues. These stromal cells may experience an inflammatory milieu, because an increase of CXCR3 was also been observed in PIN samples (data not shown). In cancer samples, mononuclear cell infiltrate may also induce CXCR3 upregulation to some extent. Endothelial cells are known to have only CXCR3B expression, which plays a role in anti-angiogenesis [18], suggesting that upregulation of CXCR3 in endothelial cells in prostate cancer could be used to limit cancer angiogenesis. More important and novel in-vivo finding from out studies was the obvious switch of CXCR3 splicing isoform in prostate cancer. The observation that CXCR3A was upregulated and CXCR3B was downregulated in localized and metastatic prostate cancer compared to normal prostate tissues suggested that this switch was not only observed in tissue-derived cell lines but also the reality in cancer samples, which may account for prostate cancer dissemination, invasion and metastasis.

Several study in animal model have reported using a CXCR3 antagonist, AMG487 or knockdown of CXCR3 to inhibit breast, colon, osteosarcoma and melanoma cell metastasis [31,34-37]. In addition, Cambien et al. recently showed that AMG487 effectively blocked colorectal cancer dissemination to lung but not to liver [38], suggesting that in vivo CXCR3-promoted cancer metastasis could be organ selective. In these studies it is not clear whether there is also a switch in CXCR3 splicing variants. Based on our cellular research, we believe AMG487 or siRNA mainly inhibit CXCR3 pro-migratory function by blocking CXCRA pathways which could be a main signaling transduction in cancer. However, since CXCR3B accounts for anti-migratory and also anti-angiogenesis signals, it would be preferential to target CXCR3A and not both isoforms.

In conclusion, our data suggest that prostate cancers subvert a 'stop' signal into a progression signal by regulating CXCR3 splicing. This is not a complete switch but a quantitative realignment. When we increase the balance of primary CXCR3 splice variant back towards CXCR3B in prostate cancer cells (DU-145), the stop signal is retained. Therefore, expression pattern of CXCR3A and CXCR3B in human prostate cancer could be a biomarker for invasive prostate cancer diagnosis. More importantly, our study has implications for rationale approaches to limiting prostate cancer invasion and metastasis.

## Materials and methods

### Cell culture and Tissue Microarrays

American Type Culture Collection (ATCC) cell lines, RWPE-1, DU-145, PC-3 and LNCaP were cultured in medium recommended by the supplier. All cells were incubated at 37°C in 5% CO_2_. The Tissue Microarray was produced by the University of Pittsburgh Prostate Tumor Bank from de-identified tumor specimens consented for research at time of treatment. Use of these tissues was approved the University of Pittsburgh Institutional Review Board.

### Quantitative real-time PCR

Total RNA was extracted from cells by RNEasy Kit (Qiagen, CA) and cDNA was reverse-transcribed by QuantiTect (Qiagen, CA). SYBR Green RT-PCR was performed (Stratagene, CA) with following primers: CXCR3: forward 5'-ACACCTTCCTGCTCCACCTA-3'; reverse 5'-GTTCAGGTAGCGGTCAAAGC-3' CXCR3A: forward 5'-AGCCCAGCCATGGTCCTTGA-3'; reverse 5'-CTGTAGAGGGCTGGCAGGAA-3' CXCR3B: forward 5'-TGCCAGGCCTTTACACAGC-3'; reverse 5'-TCGGCGTCATTTAGCACTTG-3' CXCL4: forward 5'-GCGCTGAAGCTGAAGAAGAT-3'; reverse 5'-GTCCGGCCTTGATCACCT-3'; CXCL10 forward 5'-AAGGATGGACCACACAGAGG-3'; reverse 5'-AGCAGGGTCAGAACATCCAC-3'; CXCL11 forward 5'-ATGAGTGTGAAGGGCATGGC-3'; reverse 5'-TCACTGCTTTTACCCCAGGG-3'; MMP2 forward 5'AACACAGCCTTCTCCTCCTG-3'; reverse 5'- CACCTACACCAAGAACTTCC-3'; MMP9 forward 5'- CCT CGC CCT GAA CCT GAG C -3'; reverse 5'-GCTCTGAGGGGTGGACAGTG-3' and GAPDH forward 5'-GAGTCAACGGATTTGGTCGT-3'; reverse 5'-TTGATTTTGGAGGGATCTCG-3'. GAPDH was used for control and normalization.

### Cell migration assay

Cell migration was performed as previously described [18]. Cell monolayer was allowed to become quiescent in medium with 0.1% dialyzed fetal bovine serum for 16h. Then cells were scraped to make a denuded area and treated with EGF (5 nM), CXCL10/IP10 (1 μg/ml) or CXCL4/PF4 (1 μg/ml) (PeproTech, NJ) for 16 h. Photographs were taken at 0 and 16 h, and the relative distance migrated by the cells from the edges was analyzed by ImageJ.

### Cell invasion assay

Cell invasion assay was performed by BD BioCoat Growth Factor Reduced Matrigel Invasion chamber system (BD Biosciences, MA) according to the manufacture's protocol. In brief, cells were seeded in the insert as 2.5 × 10^4 ^with quiescent medium for each assay. Then chemokines were added into the bottom chamber and cells were incubated for 48 hrs. After incubation, base membrane of the chamber was cut and cells were stained by DAPI (Sigma, MO). The total number of invaded cell was counted.

### cAMP measurement

cAMP levels were assessed using a commercially available colorimetric kit (Calbiochem, NJ). In brief, 2 × 10^5 ^cells were seeded in a well of 6-well plate and quiescent for 24 hrs. Then cells were treated with 5 nM EGF or 1 μg/ml chemokines for 5 hrs, lysed and cAMP levels were measured according to the manufacture's protocol.

### Calpain activity assay

In vivo calpain activity was determined by using the membrane permeable substrate t-BOC-LM-CMAC (BOC) (Sigma, MO). In brief, cells were incubated with 1 μg/ml chemokines for 1 hr and then 25 μM BAPTA/AM (Sigma, MO) for 10 min to chelate calcium in cells to detect only m-calpain activity. The cells were further incubated with 25 μM BOC for 20 min. The cleavage of BOC by calpain was measured using a fluorescence spectrometer.

### Immunohistochemistry

Prostate normal and tumor tissue microarray (TMA) was from tissue bank, University of Pittsburgh with IRB committee approval. TMA for immunohistochemical analysis were incubated with appropriately diluted primary antibody and secondary antibody, after antigen retrieval (BioGenex, CA). Antigen staining was performed using diaminobenzidine (DAB), then counterstained with Mayer's hematoxylin.

### In situ hybridization

The oligonucleotide probe for detecting CXCR3 mRNA (5' CGT AGA AGT TGA TGT TGA AGA GGG CAC CTG 3'), CXCR3A mRNA (5' GGT GGT CAC TCA CCT CCA GGA CCA TGG 3') and CXCR3B mRNA (5'CTC TTT TGT GAT TGA GTC TGA TTT AG 3') were labeled with DIG oligonucleotide. DIG-tailed sense CXCR3, CXCR3A and CXCR3B mRNA probes were used as negative controls. In situ hybridization was performed as previously described [51]. Briefly, sections were deparaffinized, rehydrated and then hybridization was performed at appropriate temperatures for overnight. After hybridization, nonhybridized probes were removed by high stringency washes. The sections were incubated with anti-DIG-labeled antibody conjugated with alkaline phosphatase and the signal was colorized with BCIP/NBT (Roche Applied Science, Germany).

### Plasmid or siRNA transfection

Cells were seeded as 2 × 10^5 ^cells per well of 6-well plate. After 16 hrs, cells were transfected with 2 μg of pTarget-CXCR3B plasmids and 10 μl of Lipofectamine2000 (Invitrogen, CA) according to the manufacturer's protocol. After 24 hr transfection, stable clones were selected and isolated in DU-145 medium supplemented with 1000 μg/ml G418. siRNA targeting CXCR3B (Invitrogen, CA), PLCβ3 (Santa Cruz Biotechnology, CA) or control siRNA (Santa Cruz Biotechnology, CA) were delivered into the cell by the same protocol with Lipofectamine2000.

### Immunoblotting

Cells were lysed by RIPA buffer, separated on SDS PAGE, transferred to a nylon membrane, and then immunoblotted for CXCR3 (R&D Systems, MN), CXCR3B (Protein Tech, IL), CXCL4 (R&D Systems, MN), CXCL10 (PeproTech, NJ), CXCL11 (PeproTech, NJ), PLCβ3 (Santa Cruz Biotechnology, CA), or GAPDH(Sigma, MO). The blots were visualized using chemiluminescent substrate for HRP (Thermo Scientific, IL) and X-ray film processor (AFP imaging, NY).

### Statistical analysis

All experiments were performed at least three times, each in triplicate with mean ± s.d. being presented. Statistical significance was determined using the Student t-test with statistical significance assessed with a probability value less than 0.05.

## Abbreviations

cAMP: 3'-5'-cyclic adenosine monophosphate; DAG: diacylglycerol; EGF: epidermal growth factor; EMT: epithelial-mesenchymal transition; ERK: extracellular-signal-regulated kinases; IP3: inositol 1,4,5-trisphosphate; IP9: Interferon-gamma-inducible protein 9; IP10:interferon gamma-induced protein 10; MMP: matrix metalloproteinases; PF4: platelet factor 4; PIP2: phosphatidylinositol 4,5-bisphosphate; PKA: protein kinase A; PKC: protein kinase C; PLCβ: phospholipase C β.

## Competing interests

The authors declare that they have no competing interests.

## Authors' contributions

QW and AW participated in the design of the study. QW performed experiments, analyzed data, and drafted the manuscript. RJ provided tissue samples. All authors read and approved the final manuscript.

## Supplementary Material

Additional file 1 CXCR3 expression levels in prostate cancer metastases were not organ specific (*P > 0.5). The analyses were based on data shown in Figure 1B. Metastatic prostate cancer localized in different organs was grouped and graphed with percentages of CXCR3-positive cells, including 5 lymph node metastases, 4 liver metastases, 2 lung metastases and 1 adrenal metastasis. Statistical analyses were not available in comparison to adrenal group due to a small sample size. Click here for file

Additional file 2 **CXCR3 chemokine expression in normal and prostate cancer cells**. CXCR3 chemokine expression in normal and prostate cancer cells. (A) Chemokine mRNA expression in normal and prostate cancer cells. Ligand mRNA expression was normalized to GAPDH mRNA expression. Histogram represents mean values (+/-s.d.) of three separate experiments (*P < 0.05 compared to RWPE-1 cells). (B) Chemokine protein expression in normal and prostate cancer cells. Click here for file

Additional file 3 **CXCR3 localization in normal and prostate cancer cells detected by flow cytometry**. (A) Cells were collected and treated with or without PBST (PBS with 0.5% Tween-20) to permeablize cell membrane, then further stained with CXCR3 or CXCR3B antibody for flow cytomery. Black: IgG only; Red: surface CXCR3; Green: surface and intracellular CXCR3. The graphs are representative results from more than three experiments. (B) Quantitative analysis of CXCR3 and CXCR3B localization in prostate cells based on graphs showing in (A). Histogram represents mean values (+/-s.d.) of three separate experiments (*P < 0.05 compared to RWPE-1 cells). Click here for file

Additional file 4 **CXCR3-chemokine-induced cell migration was blocked by CXCR3 antibody in DU-145 cells**. Cells were treated with chemokine with or without CXCR3 blocking antibody. Cell migration was measured by the distance change (quantified by pixel) in 16 hrs. Histogram represents mean values (+/-s.d.) of three separate experiments (*P < 0.05). Click here for file

Additional file 5 **CXCR3B downregulation did not change DU-145 cell migration**. CXCR3B was knocked down by siRNA and evaluated at (A)mRNA and (B) protein expression levels. Histogram represents mean values (+/-s.d.) of three separate experiments (*P < 0.05).(C) No change of cell migration was observed after CXCR3B downregulation. Cell migration was evaluated by the distance change (quantified by pixel) in 16 hrs. Histogram represents mean values (+/-s.d.) of three separate experiments. Click here for file

Additional file 6 **m-calpain expression in prostate normal and cancer cells**. No changes of m-calpain expression were observed after chemokine treatments (A) in normal and prostate cancer cells and (B) in DU-145 and DU-145 CXCR3 overexpressing cells. Each experiment was repeated with similar results. Click here for file

Additional file 7 **PKA activity in prostate normal and cancer cells**. PKA activity in prostate cancer cells was assessed by using a commercially available PepTag Assay kit (Promega, WI). Higher PKA activity was found in prostate cancer cells than normal prostate epithelial cells. Graph represents mean values (+/-s.d.) of three separate experiments (*P < 0.05). Click here for file

Additional file 8 **MMP mRNA expression in prostate normal and cancer cells**. (A) MMP2 and MMP9 mRNA expression in prostate normal and cancer cells (*P < 0.05 compared to RWPE-1 cells). MMP mRNA expression was normalized to GAPDH mRNA expression in each cell lines. (B) MMP2 and MMP9 mRNA expression after CXCR3 chemokine treatment in prostate normal and cancer cells. The mRNA expression levels in untreated RWPE-1 cells were set as 1. Graphs represent mean values (+/-s.e.m.) of three separate experiments each in triplicate (**P < 0.05 compared to untreated within the group). Click here for file
