# Supplementary figures and images for: Altered CXCR3 isoform expression regulates prostate cancer cell migration and invasion
Source: Mol Cancer. 2012 Jan 11;11:3. doi: 10.1186/1476-4598-11-3 (PMC3320557; doi:10.1186/1476-4598-11-3)

## Slide 1
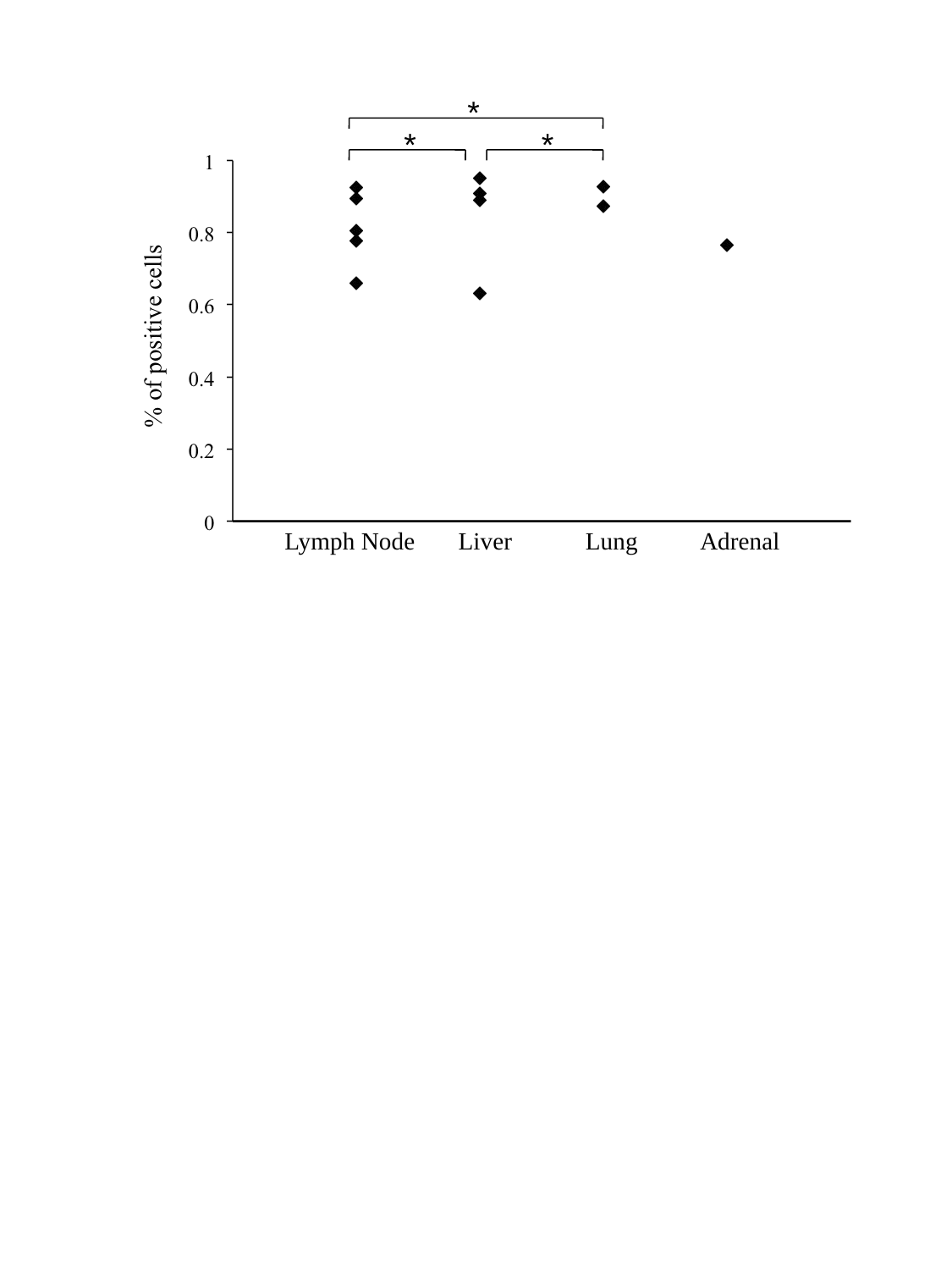

*
*
*
 Lymph Node
 Liver
 Lung
Adrenal

Supplement: Additional file 1 — CXCR3 expression levels in prostate cancer metastases were not organ specific (*P > 0.5). The analyses were based on data shown in Figure 1B. Metastatic prostate cancer localized in different organs was grouped and graphed with percentages of CXCR3-positive cells, including 5 lymph node metastases, 4 liver metastases, 2 lung metastases and 1 adrenal metastasis. Statistical analyses were not available in comparison to adrenal group due to a small sample size. [file 1476-4598-11-3-S1.PPT]

## Slide 1
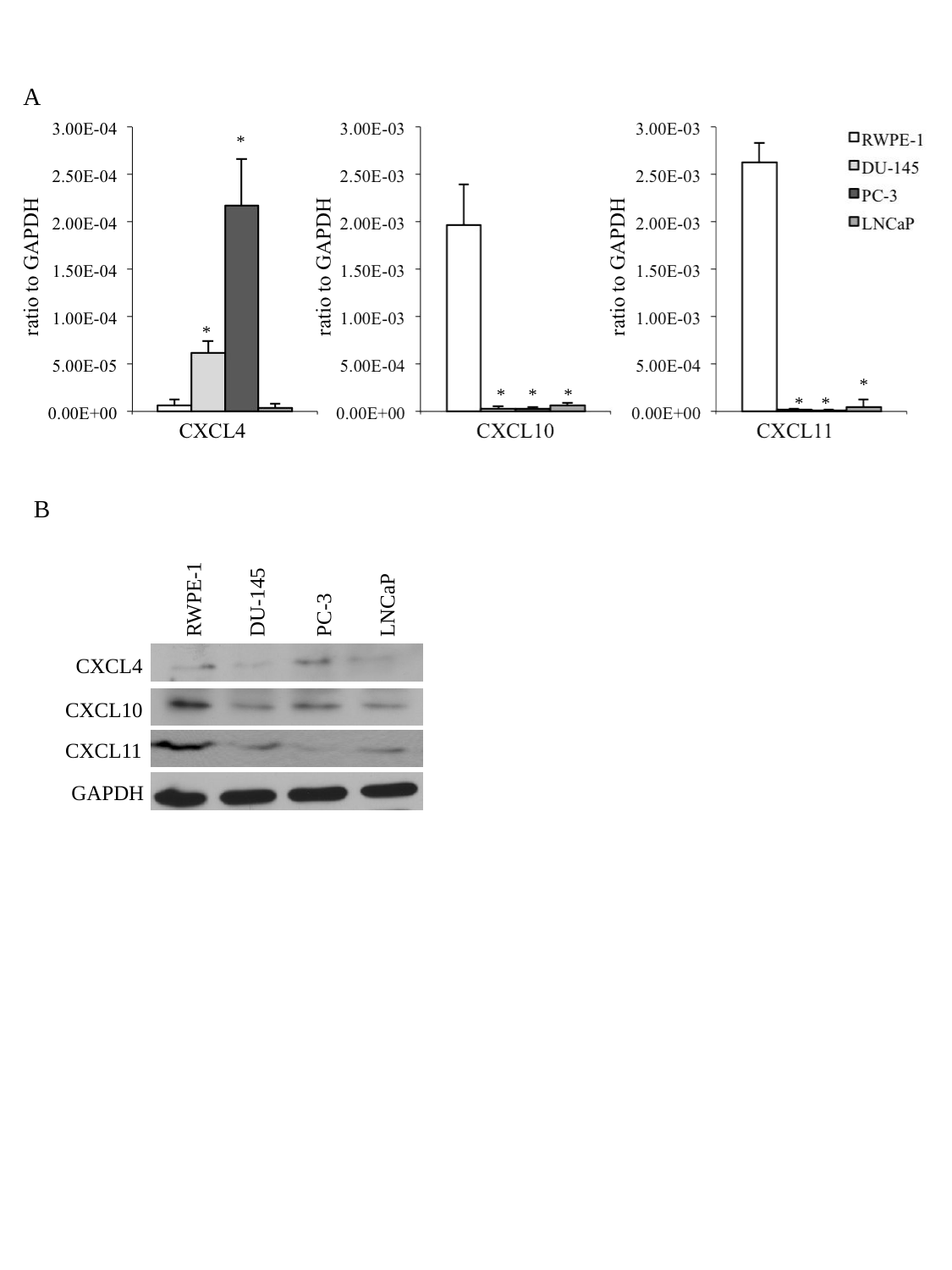

*
*
*
*
*
*
*
*
A
B
RWPE-1
DU-145
PC-3
LNCaP
CXCL4
CXCL10
CXCL11
GAPDH

Supplement: Additional file 2 — CXCR3 chemokine expression in normal and prostate cancer cells. CXCR3 chemokine expression in normal and prostate cancer cells. (A) Chemokine mRNA expression in normal and prostate cancer cells. Ligand mRNA expression was normalized to GAPDH mRNA expression. Histogram represents mean values (+/-s.d.) of three separate experiments (*P < 0.05 compared to RWPE-1 cells). (B) Chemokine protein expression in normal and prostate cancer cells. [file 1476-4598-11-3-S2.PPT]

## Slide 1
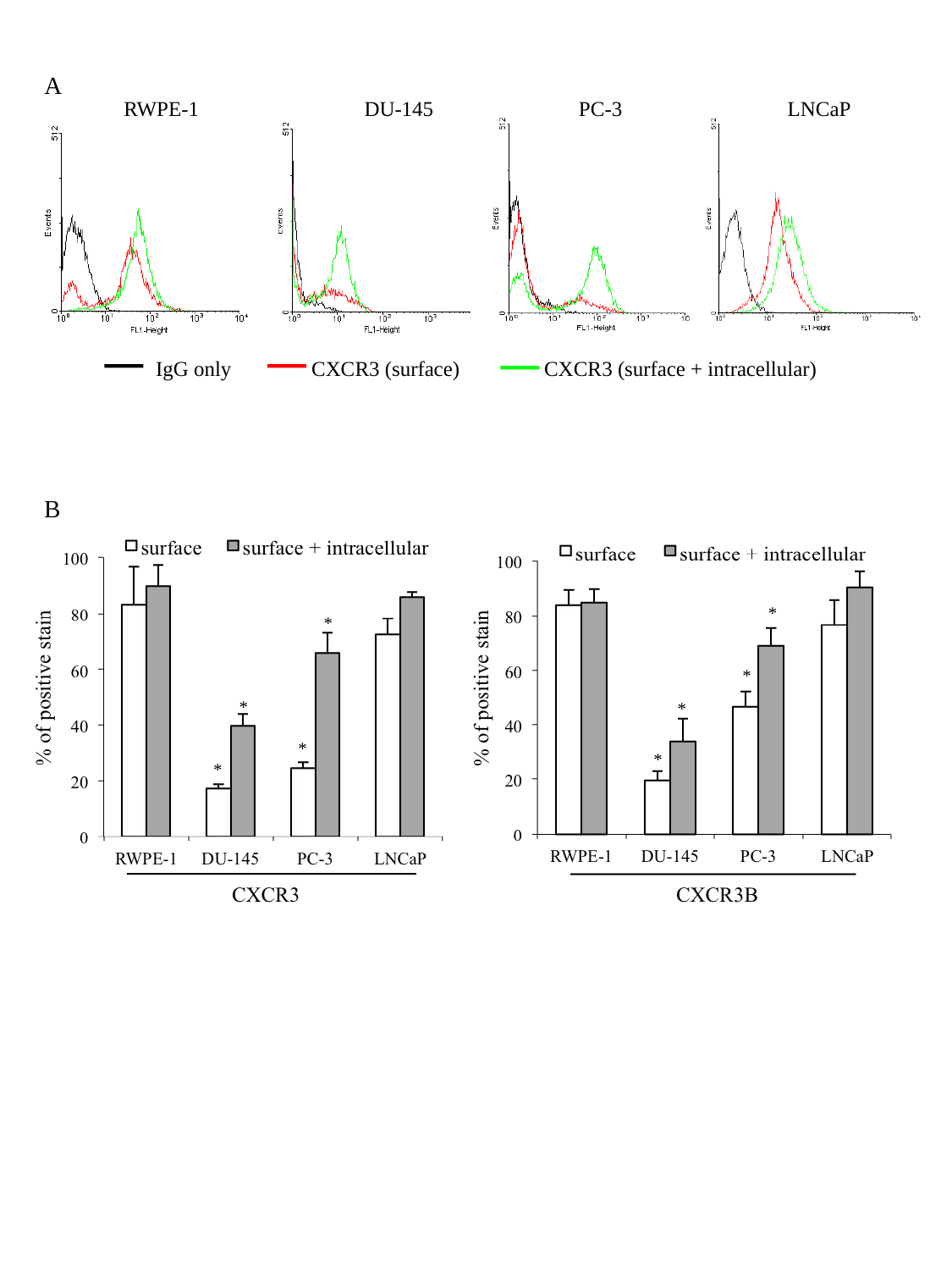

A
RWPE-1
DU-145
PC-3
LNCaP
IgG only
CXCR3 (surface)
CXCR3 (surface + intracellular)
B
 *
 *
*
 *
*
*
*
*

Supplement: Additional file 3 — CXCR3 localization in normal and prostate cancer cells detected by flow cytometry. (A) Cells were collected and treated with or without PBST (PBS with 0.5% Tween-20) to permeablize cell membrane, then further stained with CXCR3 or CXCR3B antibody for flow cytomery. Black: IgG only; Red: surface CXCR3; Green: surface and intracellular CXCR3. The graphs are representative results from more than three experiments. (B) Quantitative analysis of CXCR3 and CXCR3B localization in prostate cells based on graphs showing in (A). Histogram represents mean values (+/-s.d.) of three separate experiments (*P < 0.05 compared to RWPE-1 cells). [file 1476-4598-11-3-S3.PPT]

## Slide 1
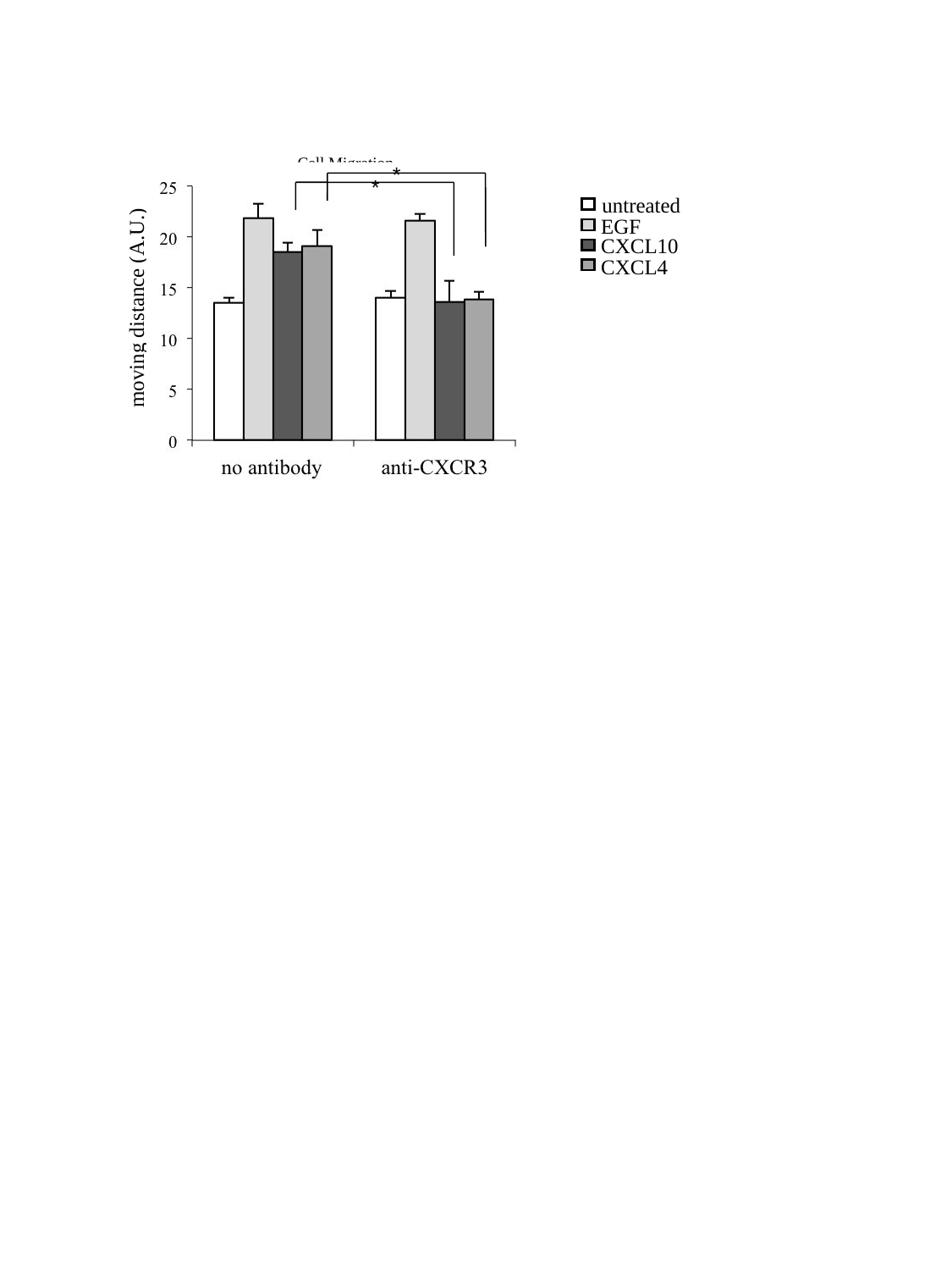

Cell Migration
*
*
moving distance (A.U.)
untreated
EGF
CXCL10
CXCL4

Supplement: Additional file 4 — CXCR3-chemokine-induced cell migration was blocked by CXCR3 antibody in DU-145 cells. Cells were treated with chemokine with or without CXCR3 blocking antibody. Cell migration was measured by the distance change (quantified by pixel) in 16 hrs. Histogram represents mean values (+/-s.d.) of three separate experiments (*P < 0.05). [file 1476-4598-11-3-S4.PPT]

## Slide 1
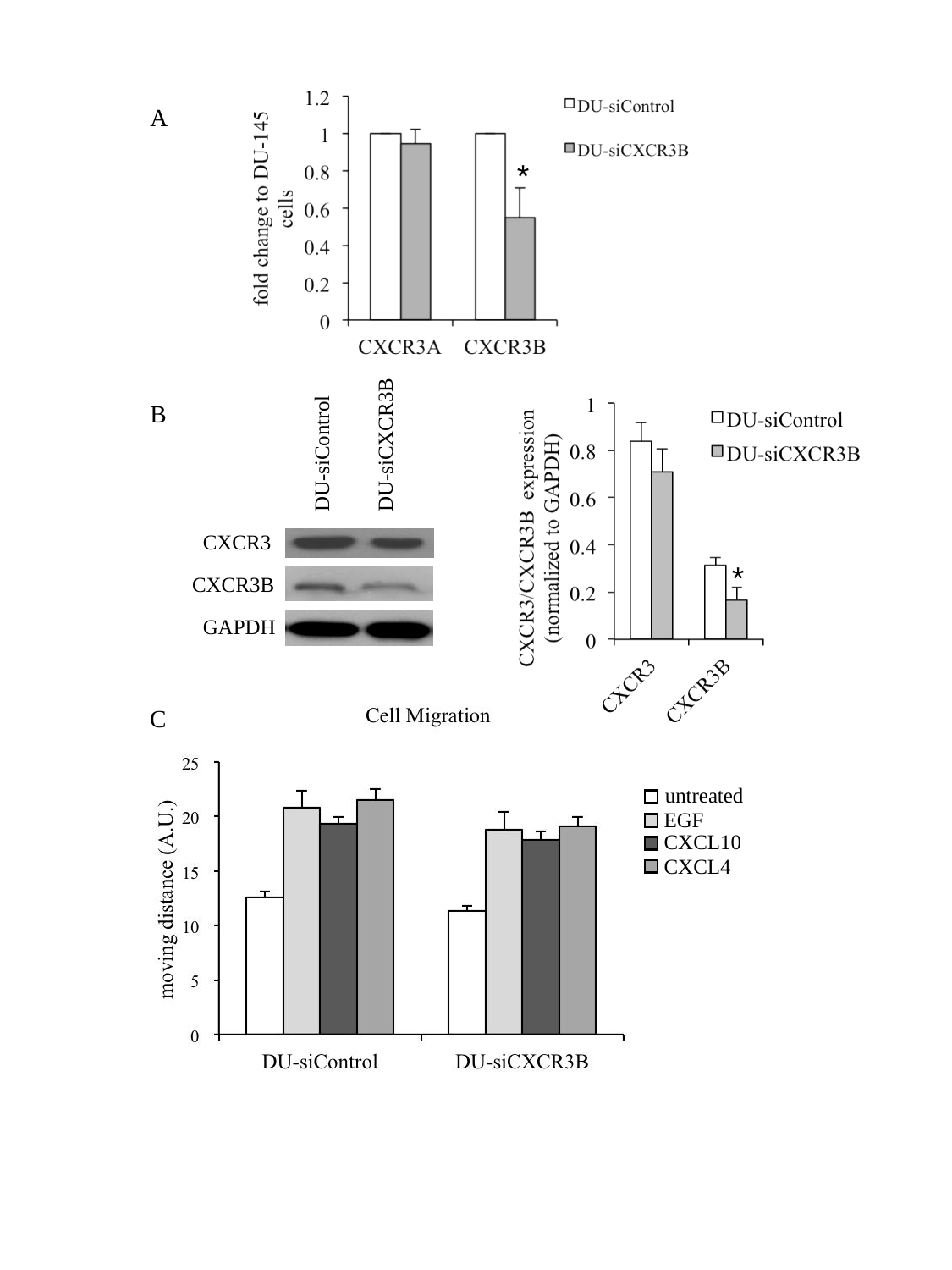

A
 *
DU-siControl
DU-siCXCR3B
B
CXCR3
CXCR3B
GAPDH
*
untreated
EGF
CXCL10
CXCL4
C

Supplement: Additional file 5 — CXCR3B downregulation did not change DU-145 cell migration. CXCR3B was knocked down by siRNA and evaluated at (A)mRNA and (B) protein expression levels. Histogram represents mean values (+/-s.d.) of three separate experiments (*P < 0.05).(C) No change of cell migration was observed after CXCR3B downregulation. Cell migration was evaluated by the distance change (quantified by pixel) in 16 hrs. Histogram represents mean values (+/-s.d.) of three separate experiments. [file 1476-4598-11-3-S5.PPT]

## Slide 1
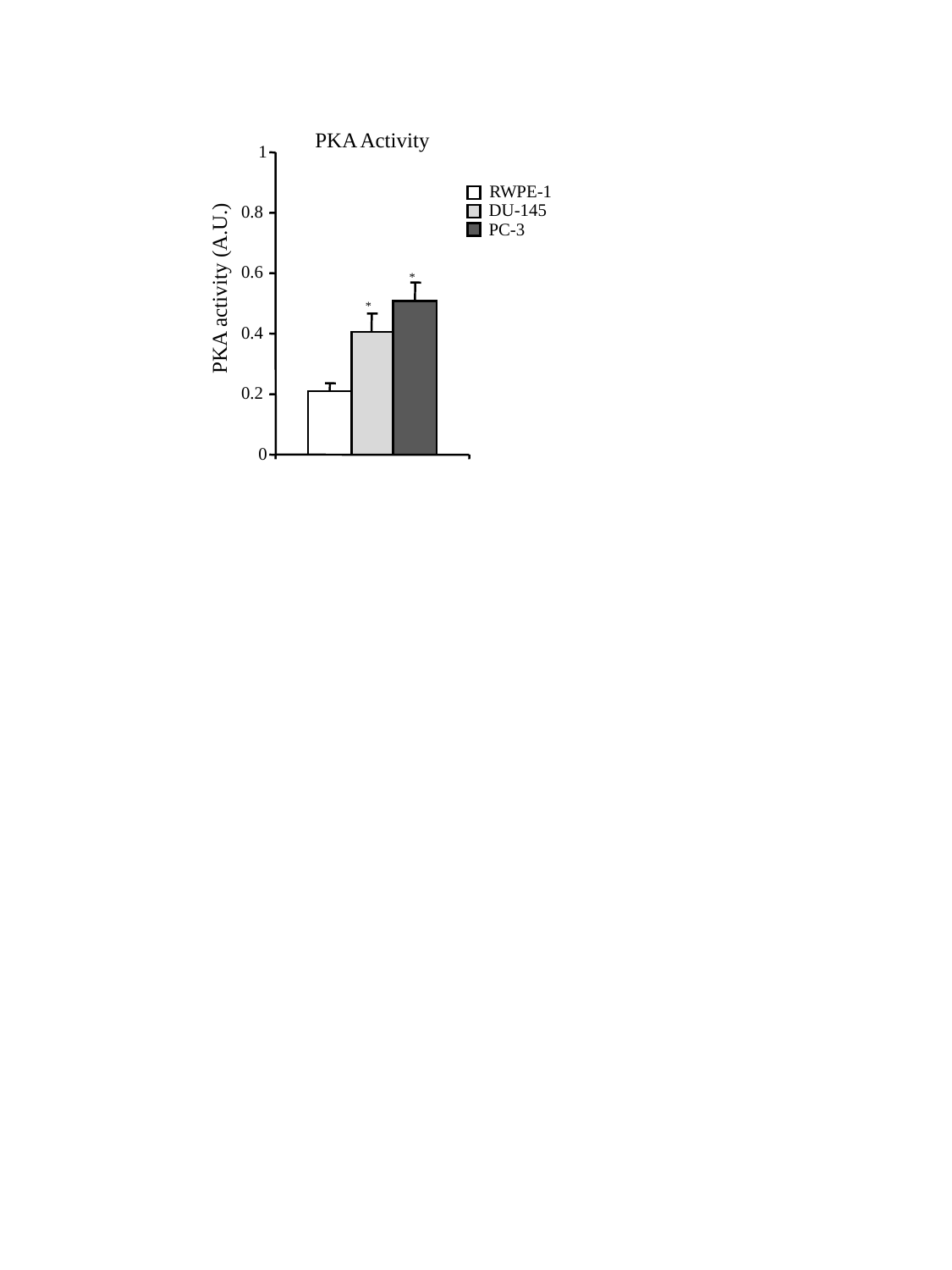

PKA Activity
1
RWPE-1
DU-145
PC-3
0.8
0.6
PKA activity (A.U.)
0.4
0.2
0
*
*

Supplement: Additional file 7 — PKA activity in prostate normal and cancer cells. PKA activity in prostate cancer cells was assessed by using a commercially available PepTag Assay kit (Promega, WI). Higher PKA activity was found in prostate cancer cells than normal prostate epithelial cells. Graph represents mean values (+/-s.d.) of three separate experiments (*P < 0.05). [file 1476-4598-11-3-S7.PPT]

## Slide 1
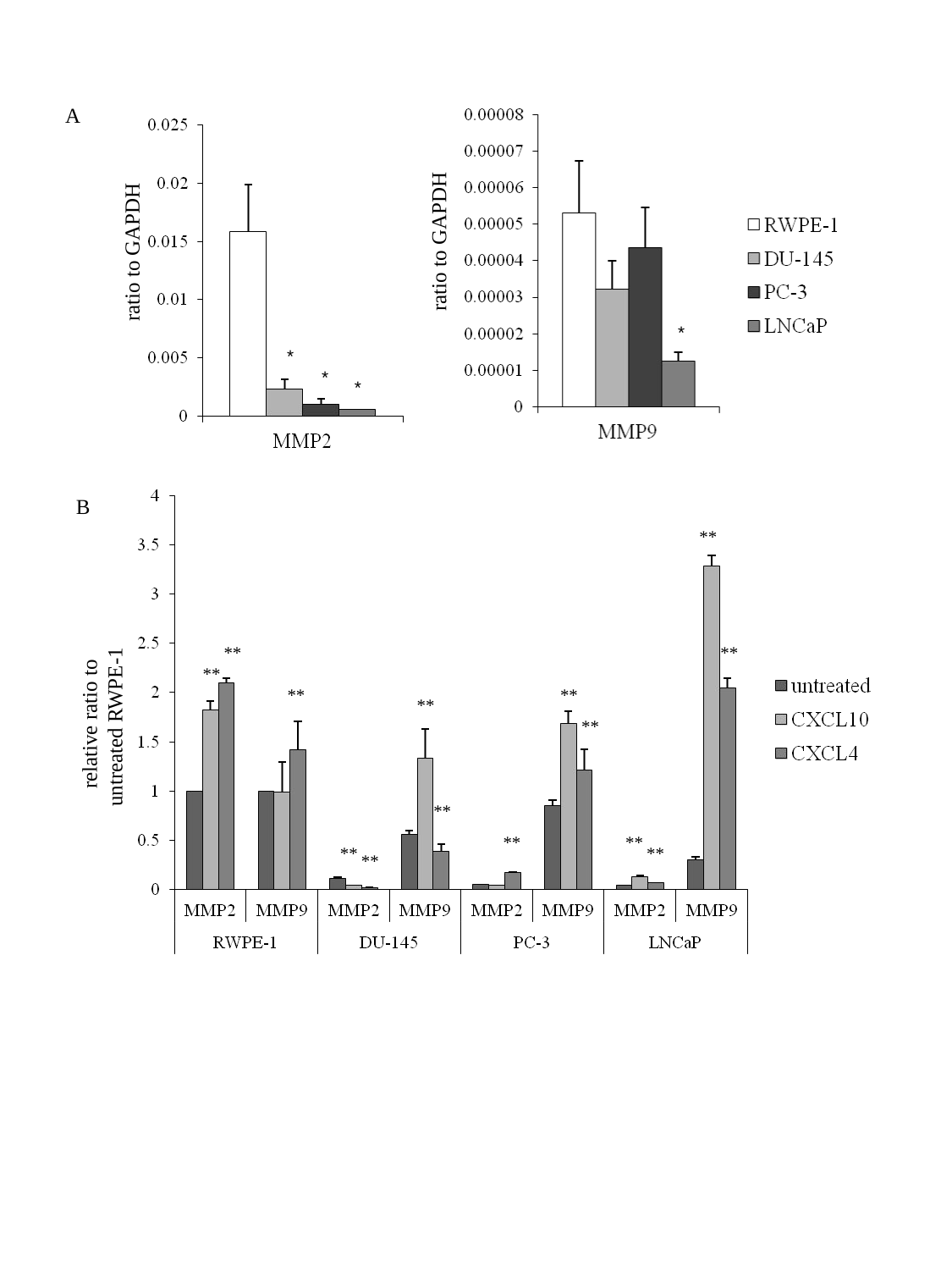

A
ratio to GAPDH
 *
ratio to GAPDH
*
*
*
**
relative ratio to untreated RWPE-1
**
**
**
**
 **
 **
 **
 **
 **
**
**
**
**
B

Supplement: Additional file 8 — MMP mRNA expression in prostate normal and cancer cells. (A) MMP2 and MMP9 mRNA expression in prostate normal and cancer cells (*P < 0.05 compared to RWPE-1 cells). MMP mRNA expression was normalized to GAPDH mRNA expression in each cell lines. (B) MMP2 and MMP9 mRNA expression after CXCR3 chemokine treatment in prostate normal and cancer cells. The mRNA expression levels in untreated RWPE-1 cells were set as 1. Graphs represent mean values (+/-s.e.m.) of three separate experiments each in triplicate (**P < 0.05 compared to untreated within the group). [file 1476-4598-11-3-S8.PPT]
