# Supplementary material for: Altered CXCR3 isoform expression regulates prostate cancer cell migration and invasion
Source: Mol Cancer. 2012 Jan 11;11:3. doi: 10.1186/1476-4598-11-3 (PMC3320557; doi:10.1186/1476-4598-11-3)
Supplement: Additional file 6 — m-calpain expression in prostate normal and cancer cells. No changes of m-calpain expression were observed after chemokine treatments (A) in normal and prostate cancer cells and (B) in DU-145 and DU-145 CXCR3 overexpressing cells. Each experiment was repeated with similar results. [file 1476-4598-11-3-S6.PPT]

## Slide 1
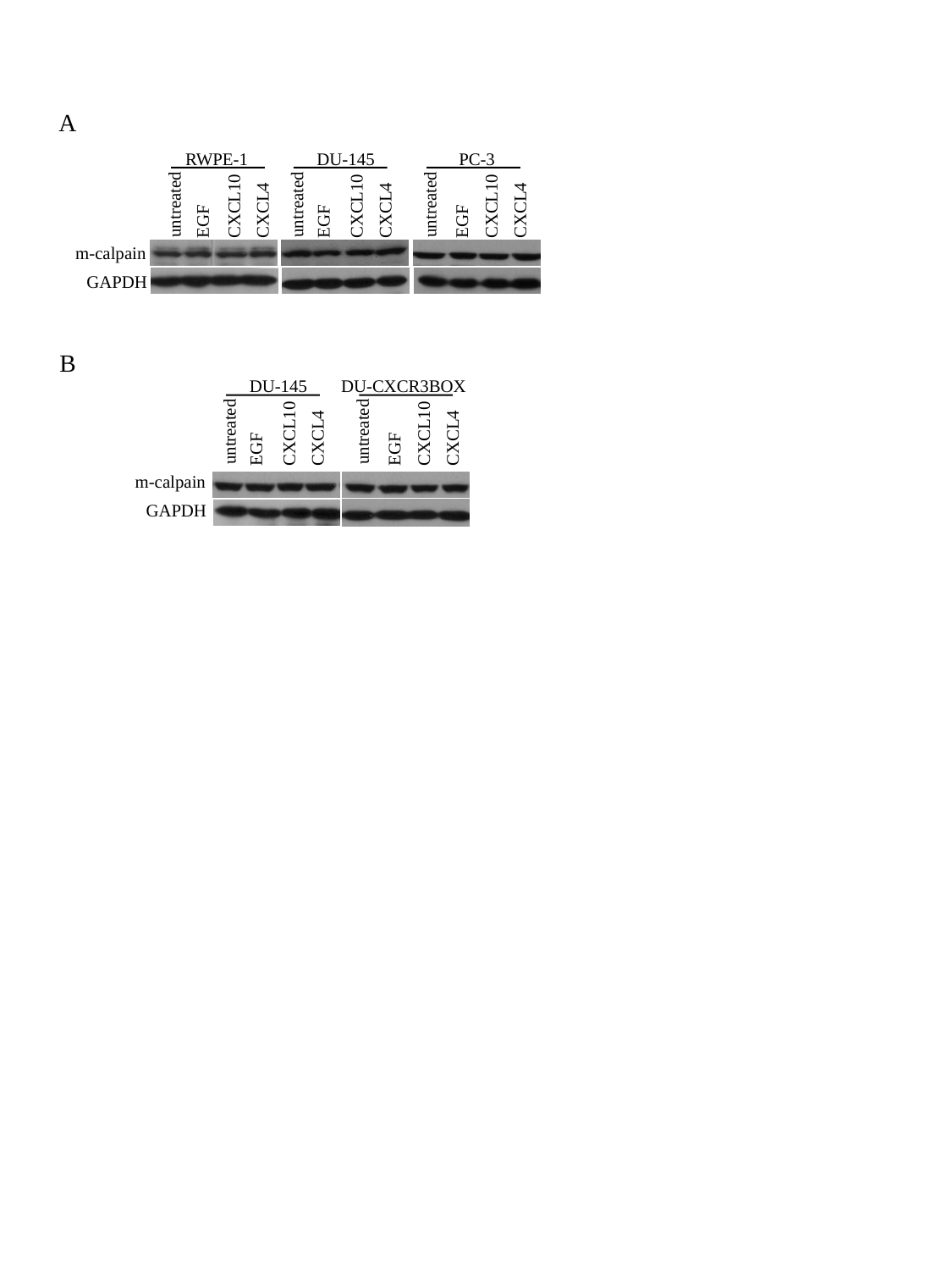

A
RWPE-1
 DU-145
 PC-3
CXCL10
CXCL4
CXCL10
CXCL4
CXCL10
CXCL4
untreated
untreated
untreated
EGF
EGF
EGF
m-calpain
GAPDH
B
 DU-145
 DU-CXCR3BOX
CXCL10
CXCL10
CXCL4
CXCL4
untreated
untreated
EGF
EGF
m-calpain
GAPDH
